# Complement Receptor Type 1 Suppresses Human B Cell Functions in SLE Patients

**DOI:** 10.1155/2016/5758192

**Published:** 2016-11-17

**Authors:** Mariann Kremlitzka, Bernadett Mácsik-Valent, Anna Polgár, Emese Kiss, Gyula Poór, Anna Erdei

**Affiliations:** ^1^MTA-ELTE Immunology Research Group, Budapest, Eötvös Loránd University, Pázmány Péter s. 1/C, Budapest 1117, Hungary; ^2^Department of Immunology, Eötvös Loránd University, Pázmány Péter s. 1/C, Budapest 1117, Hungary; ^3^Institute of Rheumatology and Physiotherapy, Frankel Leo u. 25-29, Budapest 1023, Hungary

## Abstract

Complement receptors (CRs) play an integral role in innate immunity and also function to initiate and shape the adaptive immune response. Our earlier results showed that complement receptor type 1 (CR1, CD35) is a potent inhibitor of the B cell receptor- (BCR-) induced functions of human B lymphocytes. Here we show that this inhibition occurs already at the initial steps of B cell activation since ligation of CR1 reduces the BCR-induced phosphorylation of key signaling molecules such as Syk and mitogen activated protein kinases (MAPKs). Furthermore, our data give evidence that although B lymphocytes of active systemic lupus erythematosus (SLE) patients express lower level of CR1, the inhibitory capacity of this complement receptor is still maintained and its ligand-induced clustering results in significant inhibition of the main B cell functions, similar to that found in the case of healthy individuals. Since we have found that reduced CR1 expression of SLE patients does not affect the inhibitory capacity of the receptor, our results further support the therapeutical potential of CD35 targeting the decrease of B cell activation and autoantibody production in autoimmune patients.

## 1. Introduction

The complement system is an integral part of innate immunity which provides a first-line of defence against invading pathogens [1, 2]. Apart from generating an immediate inflammatory reaction against foreign intruders, activation of complement also functions to initiate and shape the humoral immune response [3, 4]. Once activated, the complement cascade generates C3 cleavage products (C3b, iC3b, and C3d) which attach covalently to the activating agent and serve as ligands for complement receptors type 1 (CD35) and type 2 (CD21) on human B cells. CR1 binds C3b, iC3b, and C4b, possesses decay accelerating activity for the C3/C5-convertases, and serves as a cofactor for factor I-mediated cleavage of C3b [5, 6]. Although the role of mouse CR1/CR2 as a coreceptor for the BCR is relatively well established [7], differences between men and mice regarding the general structure and tissue distribution of CR1 and CR2 [8] warn us to interpret results obtained in animal studies with great care. While murine CR2 shows structural and functional homology to human CR2 and has a similar expression pattern, human CR1 is functionally different from murine CR1 and has opposite function as CR2. Our group has proven that treatment of B cells with aggregated C3, which mimics multimeric C3b and binds to CR1, strongly and dose-dependently inhibits the BCR-induced proliferation as well as antibody (Ab) production of B cells isolated from healthy individuals or rheumatoid arthritis (RA) patients [9, 10]. Likewise, cross-linking of BCR and CR1 was proven to lower the number of IgG anti-DNA producing plasma cells of lupus patients [11].

SLE is a systemic autoimmune disease characterized by dysregulation of self-reactive B cells, disturbed complement activation, and overload of immune complexes (ICs) [12]. B cells contribute to lupus pathology mainly via secretion of autoantibodies; however, other functions of B cells such as antigen (Ag) presentation and cytokine production may also be involved in the pathogenesis of SLE. Since a balanced signaling through the BCR and IC-binding coreceptors is necessary to control these B cell functions, B cell selection and activation may all be affected by altered expression and/or function of CRs [13].

Expression of CR1 on B cells has been studied in a number of human autoimmune diseases and a significant reduction was found in CR1 density compared to control subjects [14, 15]. Despite these relatively well-established changes in CR1 level of SLE patients, the functional consequences of decreased receptor expression have been studied barely. Our group revealed that although CR1 expression is markedly decreased on B cells of both active SLE and RA patients [10, 16], the inhibitory capacity of this complement receptor on RA B lymphocytes is maintained, and its ligand-induced clustering results in significant inhibition of B cell functions, similar to that found in the case of healthy individuals. This suggests that the aberrant expression of CR1 contributes to initiation of autoimmune diseases rather than altering peripheral activation of the cells [10]. The ability of human CR1 to reduce autoimmunity has also been proven in humanized SCID mice transferred with PBMCs of lupus patients where cross-linking of the BCR and CR1 restored B cell tolerance and lowered the number of IgG anti-DNA producing plasma cells [17].

Regarding the role of CR1 in regulation of B cell responses and lack of data on the functional outcome of decreased receptor expression in SLE B cells, in this study we investigated how CR1 ligation affects the BCR-driven B cell functions under physiological and autoimmune conditions. Our results demonstrate that occupation of CR1 inhibits the BCR-driven activation, proliferation, and differentiation of human B cells both in healthy individuals and in active SLE patients. Additionally, we show that CR1 clustering exerts its suppressive effect already at the initial step of B cell activation by decreasing phosphorylation of signaling molecules activated by BCR cross-linking.

## 2. Materials and Methods

### 2.1. Patients and Controls

Blood samples were obtained from 16 healthy individuals, aged 43 ± 15 years (15 women, 1 man), and from 15 active SLE patients, aged 52,8 ± 10,3 years (14 women, 1 man). Patients involved in the study met the classification criteria revised by Hochberg [18]. Patients were treated with the following drugs: nonsteroid anti-inflammatory drugs, steroid anti-inflammatory drugs (prednisolone, methylprednisolone), and disease modifying antirheumatoid drugs (methotrexate, leflunomide, chloroquine, and sulfasalazine). All patients examined for functional assays were in active disease state (average SLEDAI score: 6.4). The study was approved by the local ethical committee (Institutional Review Board of National Institute of Rheumatology and Physiotherapy) and written informed consent was obtained from all participating subjects.

### 2.2. Culturing Conditions

Peripheral B cells were isolated from healthy individuals and active SLE patients with the Pan B cell isolation Kit (Miltenyi Biotec, Germany) through negative selection. The B cell yield was >90% in each isolation. Cells were cultured at 2 × 10^5^ cells/well in 100 *μ*L RPMI-I 1640 medium (Sigma Aldrich, Hungary) supplemented with 10% FCS and gentamycin in 96-well microtiter U-bottom plates (Costar, USA) at 37°C in a humidified atmosphere containing 5% CO_2_. B cells were stimulated with 5 *μ*g/mL anti-human IgG+M+A (Jackson ImmunoResearch, Newmarket, UK) in the presence or absence of heat-aggregated C3, the C3b-like ligand of human CR1 [9, 19] or C3b (Calbiochem, coated on the plate), or the CR1 specific monoclonal antibody, To5 (Santa Cruz Biotechnology).

### 2.3. Isolation of Human C3, Generation of C3b-Like C3

Human C3 was isolated from pooled normal human serum by fast protein liquid chromatography as described by Basta and Hammer [20]. Purified C3 was collected, concentrated, and dialyzed against PBS. Traces of IgG contamination were eliminated by incubation of the C3 solution with protein G beads (Thermo Scientific, Rockford). The purity of C3 was checked by SDS-PAGE and Coomassie blue staining. C3 fractions were stored at −80°C until use. Aggregated C3 was generated by incubating isolated human C3 at 63°C for 20 min.

### 2.4. Proliferation Assay

Cell proliferation was assessed by H^3^-thymidine incorporation after 48 hours by pulsing the cells with 1 *μ*Ci/well H^3^-thymidine (NEN, Boston, MA) for the last 18 hours of culture. Incorporated radioactivity was measured with a Wallac 1409 liquid scintillation beta counter (Wallac, Allerod, Denmark). Results are expressed as mean cpm ± SD of triplicate samples or mean % of inhibition ± SEM of five independent experiments.

### 2.5. Measurement of B Cell Activation

Activation of B cells was monitored through changes in the expression of CD69 and CD40 after 48 hours by flow cytometry as described earlier. Briefly, activated B cells were washed in PBS and were stained with FITC-conjugated anti-CD69 (BD Biosciences) and APC-conjugated CD40 (Immunotools, Friesoythe, Germany) antibodies according to the manufacturer's instruction. After incubation on ice for 30 minutes, cells were washed and resuspended in 200 *μ*L PBS containing 1% FCS and 0.1% NaN_3_. Data from 20.000 cells were collected using a FACSCalibur flow cytometer and the CellQuest software (BD Biosciences). Results are expressed as mean fluorescence intensity (MFI) ± SD of duplicate samples or mean % of inhibition ± SEM of five (SLE patients) or nine (healthy controls) independent experiments.

### 2.6. B Cell Plasmablast Differentiation Assay

To induce plasmablast differentiation and Ig production, B cells were activated with 5 *μ*g/mL anti-human IgG+M+A (Jackson) in the presence of 50 ng/mL recombinant human (rh) IL-2, 50 ng/mL rh IL-10, and 50 ng/mL rh IL-6. After 6 days, the cells were used to determine the number of CD38^+^CD19^low^CD27^high^ plasmablasts by flow cytometry, and culture supernatants were collected to analyze the amount of secreted IgM by chip method. Briefly, cells were washed in ice-cold PBS and then stained with the following antibodies: anti-human CD38-FITC (Immunotools), anti-human CD27-PE (Caltag Laboratories, Bangkok, Thailand), and anti-human CD19-APC (Immunotools). Measurements were performed using a FACSCalibur flow cytometer (BD Biosciences). Data of 100.000 cells were collected and % of plasmablasts was counted in the living B cell population. Data presented show mean % of plasmablasts ± SD of duplicate samples or mean % of inhibition ± SEM of six independent experiments.

### 2.7. Assay for Ig Production

IgM production of isolated human B cells was measured by protein microarray. Anti-human IgM were printed in triplicate of 2 different dilutions onto nitrocellulose-covered glass slides using a BioOdyssey Calligrapherminiarrayer (Bio-Rad Laboratories, Hercules, CA, US). Arrays were rinsed in PBS for 15 minutes before use and incubated with 100 *μ*L cell culture supernatants of plasmablast assays for 1 hour at 37°C. Following washing in PBS containing 0.05% Tween, microarrays were incubated with DyLight 488-conjugated F(ab')_2_ fragment of goat anti-human IgM (Jackson) (1 : 2500, diluted in BSA-PBS-Tween) for 30 minutes. After washing in PBS-Tween, arrays were dried and scanned with an Axon GenePix 4300A scanner and data were analyzed with GenePix Pro 7 software (Molecular Devices). Relative fluorescence intensities (RI) were calculated by subtracting background fluorescence from the median of three parallel signal intensities. Signals not exceeding two standard deviations of local background signals were clamped to an arbitrary value of 1.

### 2.8. Phosphokinase Specific Flow Cytometry (Phosphoflow Assay)

10^6^ PBMCs/sample were activated with 5 ug/mL anti-human IgG+M+A for 10 minutes in the presence or absence of aggregated C3 at 37°C. After treatment, PBMCs were labelled with anti-human CD19-FITC (Immunotools) for 25 minutes at 4°C and fixed with 2% paraformaldehyde for 10 minutes at 37°C. The fixed PBMCs were then permeabilized on ice for 30 minutes using 0.5 mL 100% methanol (Reanal, Budapest, Hungary). Cells were then washed twice and stained with anti-phospho-Syk (p-Syk, sc-293118, Santa Cruz Biotechnology), anti-phospho-p44/42 MAPK (p-ERK1/2, #4370), or anti-phospho-SAPK/JNK (p-JNK, #4668). As secondary Ab, AF 647-conjugated goat anti-rabbit Igs (Invitrogen, MA, US) were used. The expression of phosphorylated signaling molecules was analyzed by flow cytometry (FACSCalibur). Dot plots presented show percentage (%) of p-Syk, p-ERK, or P-JNK positive cells and are representative of three independent experiments. Graphs illustrate mean % of inhibition ± SEM of three independent experiments.

### 2.9. Statistical Analysis

CR1-mediated inhibition was calculated by dividing the value of activated and CR1-agonist (either aggregated C3 or C3b or anti-CR1 Ab) treated samples with that of the activated but CR1-agonist untreated samples (i.e., when only anti-human IgG+A+M stimulus was employed). Statistical differences were assessed by Permutation-test using R-statistic and figures were prepared with PrismSoftware, version 4.0 (GraphPad Software). Values of *P* < 0.05 were considered statistically significant.

## 3. Results

### 3.1. Ligation of CR1 Inhibits BCR-Induced Proliferation of B Cells Derived from Healthy Humans and Active SLE Patients

**Figure 1:**
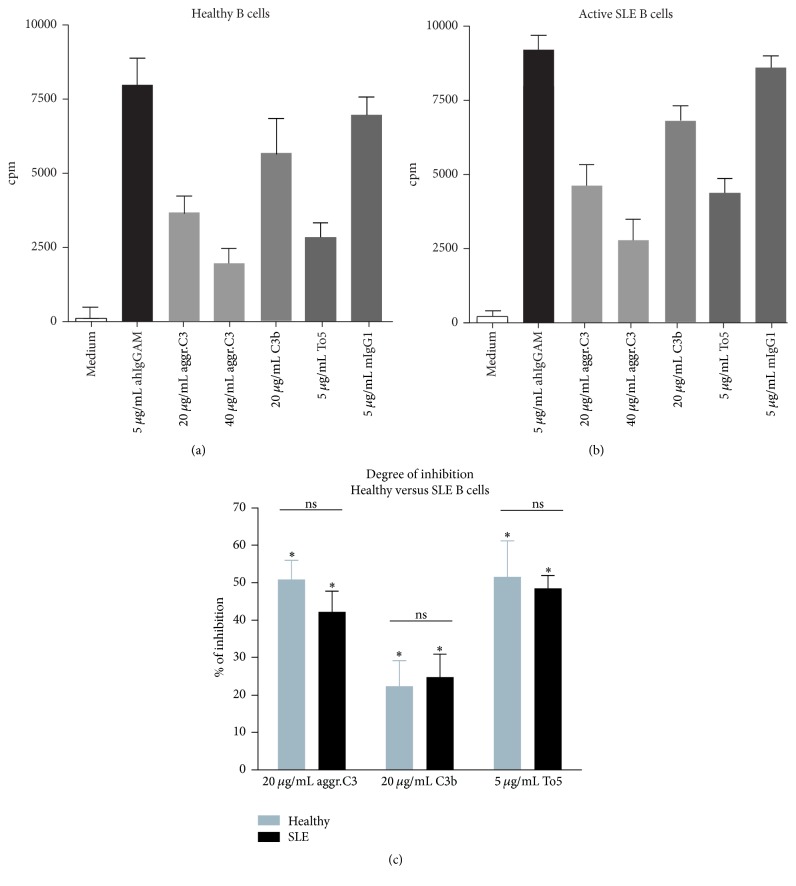
Impact of CR1 clustering on BCR-induced proliferation of B cells. 2 × 10^5^ B cells isolated from healthy donors (a) or active SLE patients (b) were activated with 5 *μ*g/mL F(ab')_2_ fragment of anti-human IgG+M+A Ab in the presence or absence of different concentrations of heat-aggregated C3, surface coated C3b, or the CR1-specific antibody (To5) clustered by anti-mouse IgG. As control, cells were cultured in medium or with an isotype-matched control mouse IgG. Cells were harvested after pulsing with 1 *μ*Ci/well H^3^-thymidine for the last 16 hours of culture. (a and b) Data shown are mean ± SD cpm of triplicate cultures and representative of five independent experiments with similar results is shown. (c) Results showing percent of inhibition mediated by aggregated C3, C3b, or the anti-CR1 Ab (To5) are mean % of inhibition ± SEM of five independent experiments and correlated with the CR1-agonist untreated sample (see Section 2, Permutation-test; ^*∗*^ *P* < 0.05 and ^ns^ *P* > 0.05).

We described earlier that clustering of CR1 via its ligand inhibits BCR-induced activation of B cells obtained from healthy individuals [9]. However, it has not been investigated so far how decreased CR1 level affects the physiological inhibitory function of the receptor in SLE patients. To evaluate the potential modulatory effect of reduced receptor expression, isolated B cells of healthy individuals or active SLE patients were activated via the BCR and CR1 was targeted simultaneously either by the well-characterized “C3b-like” agonist, aggregated C3 [9, 10] or the natural ligand, C3b or the CR1-specific monoclonal Ab, To5. As seen in Figure 1(a), both C3b and the C3b-like ligand, aggregated C3, exerted a significant inhibition on the BCR-induced proliferation of B cells of healthy donors. The Ab-mediated cross-linking of CR1 had a similar effect as the multimeric ligand; namely, it caused a strong inhibition of B cell proliferation. Strikingly, as illustrated in Figure 1(b), ligation of CR1 reduced the proliferation of SLE B cells too, similar to healthy controls. These data demonstrate that although CR1 expression in SLE patients is decreased, this reduction does not affect the physiological inhibitory capacity of the receptor and the degree of inhibition is similar in case of both healthy and autoimmune B cells (Figure 1(c)).

### 3.2. CR1 Inhibits the BCR-Driven Upregulation of Activation Markers on B Cells of Healthy Humans and Active SLE Patients

**Figure 2:**
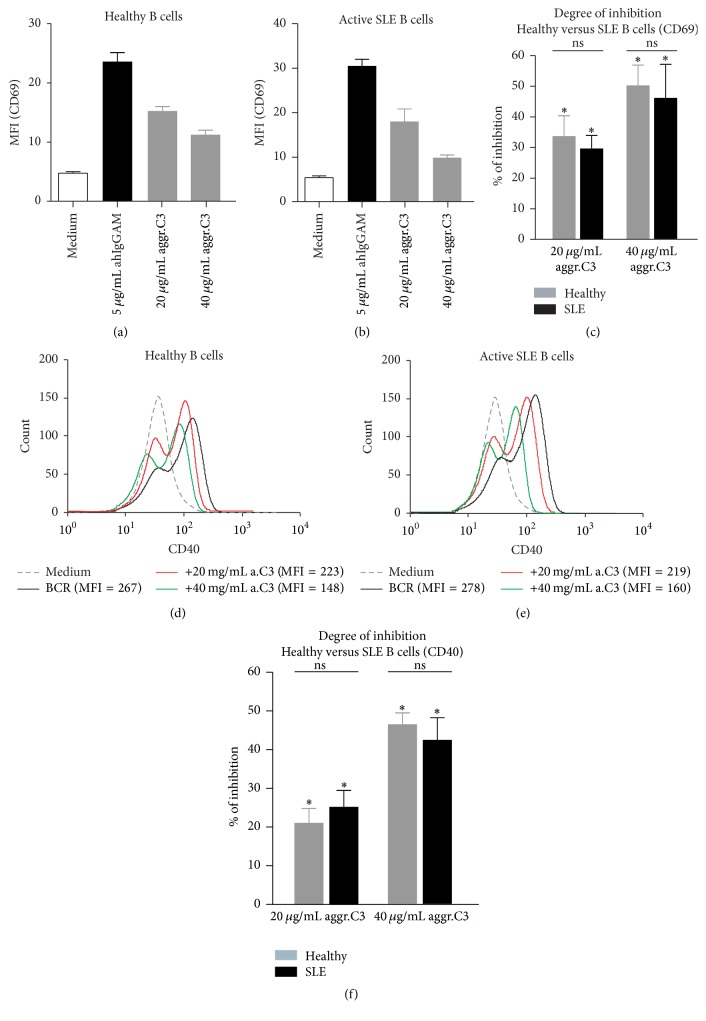
Effect of CR1 clustering on BCR-induced upregulation of activation markers in healthy controls and active SLE patients. 2 × 10^5^ B cells isolated from healthy donors (a and b) or active SLE patients (d and e) were activated for 48 hours with 5 *μ*g/mL F(ab')_2_ fragment of anti-human IgG+M+A in the presence or absence of different concentrations of heat-aggregated C3. As control, cells were left untreated. Results shown are either flow cytometric histograms or mean ± SD geometric mean fluorescence intensity (gMFI) of duplicate cultures of CD69 (a and b) and CD40 (d and e) expression and are representative of nine (healthy controls) or five (SLE patients) independent experiments. (c and f) Results showing percent of inhibition are mean % of inhibition ± SEM of nine (healthy controls) or five (SLE patients) independent experiments and correlated with the CR1-agonist untreated sample (see Section 2, Permutation-test; ^*∗*^ *P* < 0.05 and ^ns^ *P* > 0.05).

Activation of B cells induces upregulation of costimulatory molecules and enhances Ag presentation to T cells [21]. Therefore, we went on to test the effect of CR1 ligation on the BCR-induced changes in the phenotype of B lymphocytes. To this end, isolated B cells were activated via the BCR in the presence or absence of the CR1 ligand and changes in the expression of CD40 and CD69 were monitored after 2 days. As shown in Figure 2, clustering of CR1 by the C3b-like ligand dose-dependently inhibited the BCR-induced upregulation of the activation marker, CD69 (Figure 2(a)), and the costimulatory molecule, CD40 (Figure 2(d)), on B cells of healthy individuals. Again, the lower expression of CR1 in SLE patients did not influence the inhibitory function of the receptor (Figures 2(b) and 2(e)) and the extent of reduction of activation markers was similar to that found in healthy subjects (Figures 2(c) and 2(f)).

### 3.3. Effect of CR1 Clustering on BCR-Induced Plasmablast Formation and IgM Production

**Figure 3:**
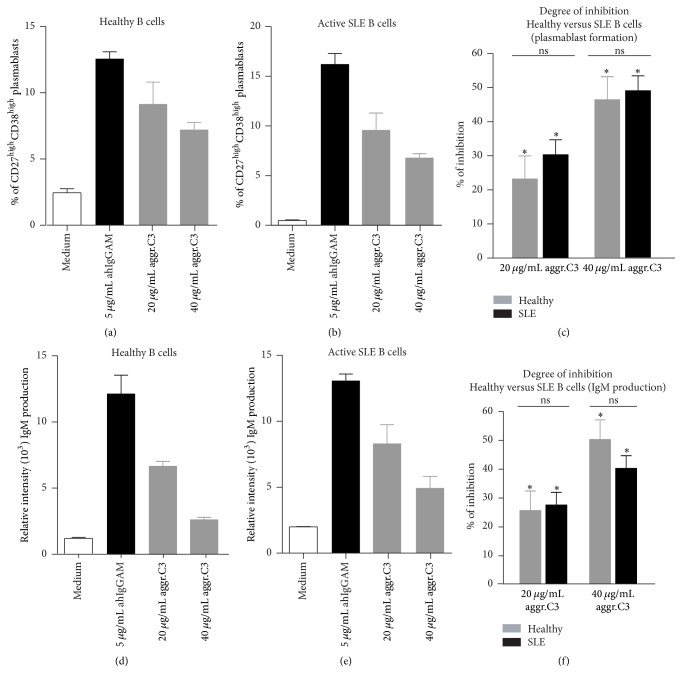
Impact of CR1 clustering on BCR-induced plasmablast formation (a–c) and IgM secretion (d-e) of human B cells. 2 × 10^5^ B cells isolated from healthy donors (a and d) or active SLE patients (b and e) were activated for 6 days with the F(ab')_2_ fragment of anti-human IgG+M+A Ab (5 *μ*g/mL) in the presence of 50 ng/mL IL-10, IL-2, and IL-6 and treated with different concentrations of heat-aggregated C3. (a and b) The percentage of CD19^low^CD27^high^CD38^high^ plasmablasts was determined at day 6 by flow cytometry. Data show mean frequency of CD19^low^CD27^high^CD38^high^ cells ± SD of duplicate samples. One representative experiment of six is shown. (d and e) Supernatants of cultured cells were collected on day 6 and were measured for secreted IgM by reverse phase protein microarray. Data are expressed as mean ± SD relative fluorescence intensity of triplicate measurements. Results are representative of six independent experiments in the case of both healthy donors (d) and active SLE patients (e). (c and f) Results showing percent of inhibition mediated by aggregated C3 are mean % of inhibition ± SEM of six independent experiments and correlated with the CR1-agonist untreated sample (see Section 2, Permutation-test; ^*∗*^ *P* < 0.05 and ^ns^ *P* > 0.05).

In the next step we set out to investigate whether CR1 cross-linking also affects the differentiation of B cells into Ab-secreting plasmablasts and their subsequent IgM production. To this end, B cells were activated for 6 days via their BCR with 5 *μ*g/mL anti-human IgG+M+A in the presence of IL-2, IL-6, and IL-10 with or without aggregated C3. After 6 days, B lymphocytes were washed and stained for flow cytometry to assess the number of CD19^low^CD27^high^CD38^high^ plasmablasts. As seen in Figures 3(a) and 3(b), B cells cultured only in the presence of cytokines contained very few CD19^low^CD27^high^CD38^high^ plasmablasts (ranging between 0.5 and 3%). Stimulation with anti-human IgG+M+A caused an elevation in the number of plasmablasts (ranging between 5 and 15%) which was dose-dependently inhibited by CR1 clustering both in healthy individuals (Figure 3(a)) and in active SLE patients (Figure 3(b)). This demonstrates again that the altered receptor expression does not influence the physiological inhibitory function of CR1 (Figure 3(c)).

Since we found that B cell differentiation to plasmablast is strongly inhibited by CR1, we supposed that the major B cell function, namely, Ig production, is also affected by the complement-derived ligand. To assess this, supernatants of B cells cultured as described in the previous paragraph were collected at day 6 and measured for the amount of secreted IgM. We found that BCR-induced plasmablast formation of healthy B cells was accompanied by an increased production of total IgM which was decreased by CR1 clustering both in healthy controls (Figure 3(d)) and in active SLE patients (Figure 3(e)). Again, we found no statistically significant difference in the degree of inhibition between healthy donors and autoimmune patients (Figure 3(f)).

### 3.4. Phosphorylation of Key Signaling Molecules of the BCR Pathway Is Inhibited by CR1 Ligation

**Figure 4:**
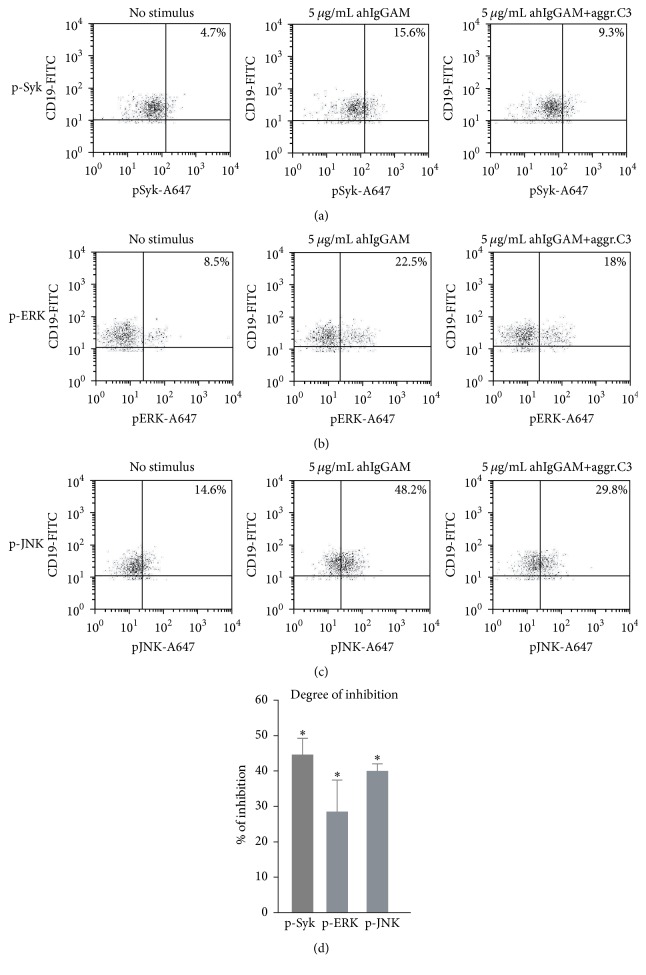
Effect of CR1 clustering on BCR-induced phosphorylation of Syk and MAPKs in human B cells. (a) 10^6^ PBMCs isolated from healthy donors were activated for 10 minutes with 5 *μ*g/mL F(ab')_2_ fragment of anti-human IgG+M+A and treated or not with 40 *μ*g/mL heat-aggregated C3. Effect of CR1 clustering on phosphorylation of Syk (a), ERK (b), and JNK (c) was investigated by the Phosphoflow method. Dot plots show percentage (%) of p-Syk, p-ERK, or p-JNK positive B cells and are representative of three independent experiments with similar results. (d) Results showing percent of inhibition mediated by aggregated C3 are mean % of inhibition ± SEM of three independent experiments and correlated with the CR1-agonist untreated sample (see Section 2, Permutation-test; ^*∗*^ *P* < 0.05 and ^ns^ *P* > 0.05).

To find out at which level of the signaling cascade CR1 exerts its inhibitory function, B cells were activated via their BCR and treated with the C3b-like ligand, aggregated C3. Changes in the phosphorylation of key signaling molecules were investigated by the Phosphoflow method. As seen in Figure 4, the phosphorylation of mitogen activated protein kinases (MAPKs), such as ERK and c-JunN-terminal kinase (JNK) in BCR-activated B cells, was significantly reduced after CR1 clustering. To test whether CR1 influences the BCR-induced signaling at an earlier step of activation, we also monitored how CR1 ligation affects phosphorylation of the membrane proximal kinase, Syk. Our results show that the C3b-like ligand strongly reduces the BCR-induced phosphorylation of Syk, highlighting that the receptor exerts its inhibitory effect already at the initial steps of B cell stimulation. Due to reduced availability of patient samples, Phosphoflow experiments could be carried out only once in SLE B cells; however, similar to the functional assays, CR1 ligation reduced the BCR-induced activation of Syk and the selected MAPKs in the autoimmune patients too (data not shown).

## 4. Discussion

The complement system is known to play a key role in priming and regulating the adaptive immune response. The adjuvant property of CR2 in B cell activation and Ab production via recognition of C3d(g) opsonized antigens has been thoroughly studied in the last decades mainly in mice [3, 7]. In contrast to this, the role of CR1 in regulation of human B cell functions is poorly understood. Here we provide evidence that CR1 has opposite function in human B cells than CR2 since it inhibits the BCR-driven proliferation (Figure 1), upregulation of costimulatory molecules (Figure 2), and Ab secretion (Figure 4) under both physiological and autoimmune conditions.

The CR1-mediated inhibition of B cell functions described here may provide an additional regulatory level for the humoral immune response during infections. Pathogens invading our body are likely to activate complement and “innate-like” B cells at the same time, causing inflammation, opsonization, and killing of the pathogen. Generation of such inflammatory environment has a clear advantage in elimination of infections; however, an excessive activation, under pathological conditions, may be harmful for the host. Therefore, complement activation and B cell priming need to be tightly controlled and inhibited at different points. CR1 belongs to the family of complement inhibitors acting as a cofactor for the Factor I-mediated cleavage of C3b and exerting decay accelerating activity for C3- and C5-convertases. Our results give evidence that, beside these classical regulatory functions, binding of C3b- and C4b-coated ligands to CR1 also decreases the early, polyclonal activation of B cells (mimicked by BCR stimulation here), providing an additional brake for the initiation of protective immunity. This phenomenon has a clear advantage from an evolutionary point of view since complement activation not only generates an acute inflammation to eliminate infection but also prevents overreaction of the host immune system by inhibition of noncognate B cell priming. Nevertheless, this low level of inhibition may still allow further spreading of the more specific humoral immune response and endows the innate signals to focus only on Ag-stimulated B cells.

Systemic autoimmune diseases are frequently associated with the production of autoantibodies raised against nuclear components of dying cells. Association of these antibodies to self-antigens and complement factors will form circulating ICs which are likely one of the source materials for autoreactive B cell activation in SLE. Although self-reactive B cells that escape negative selection often express low-affinity receptors for self-Ags, they can be activated by integrating stimulatory signals from additional receptors. For instance, nucleic acid-protein complexes are thought to activate B cells via simultaneous ligation of the BCR and Toll-like receptors, leading to secretion of autoantibodies and proinflammatory cytokines [22, 23]. In contrast to this, no data are available how concurrent ligation of complement receptors and BCR, via binding of complement containing ICs, affects the final outcome of B cell activation in SLE patients.

We have found that, despite the earlier described reduction in CR1 expression of SLE patients, its inhibitory capacity is still preserved. We show that binding of the C3b-like ligand results in a significantly reduced B cell proliferation (Figure 1(b)), upregulation of costimulatory molecules (Figure 2(e)), and antibody secretion (Figure 3(e)), probably by reducing phosphorylation of key signaling molecules of the BCR-induced signaling cascade (Figure 4). Assessing the function of the activatory CR2 in the case of SLE patients, Mitchell et al. also found that, despite the fact that B cells were found to express half as many surface CR2 as normal B lymphocytes, the Ca^2+^ response and the percentage of responding cells were significantly increased after coligation of BCR and CR2 [24]. Thus, it seems that the altered expression of CR1 and CR2 on peripheral B cells of SLE patients does not affect the physiological function of the receptors.

One of the explanations of this phenomenon might be that at suboptimal Ag concentration, as employed in our experiments, B cells of SLE patients still express enough CR1 to inhibit BCR-induced responses. However, persistent stimulation by abundantly generated ICs, as seen in chronic autoimmune diseases, might overcome this inhibitory capacity and B cells could be inappropriately activated. Furthermore, no data are available about the signaling mechanism of CR1 in human B cells. CR2 appears mainly complex with CD19 which is the signaling partner of CR2. Although the level of CR2 is decreased in B cells of SLE patients, the comparable expression of CD19 in autoimmune and control B cells might explain the normal response of patients to cross-linking of CR2 and IgD [24] and the same phenomenon might operate in the case of CR1. Therefore, we propose that the reduced expression of CR1 and CR2 affects receptor functions only when their level falls below a critical density and only the combined alteration in receptor expressions may be sufficient to cause dysregulation of B cell functions.

Alternatively, downregulation of complement receptors on B cells may contribute to the initiation of autoimmunity by affecting B cell selection rather than influencing disease severity itself. Several animal models suggest that CR1 and CR2 play an indispensable role in the development of tolerance by presentation of self-antigens to autoreactive B cells at the immature stage [25]. At this stage encounter of B cells with autoantigens results in negative selection; however, defect in retention of self-antigens due to abnormal expression of CR1 and CR2 may lead to loss of deletion and escape of anergic, self-reactive B cells to the periphery. This hypothesis is supported by the fact that CD21^low/−^ B lymphocytes have been found enriched in patients with autoimmune diseases [26, 27] and these cells also fail to express CD35 (our unpublished results). CD21^low/−^ B cells are refractory to most stimulation suggesting that they use an anergic mechanism to be tolerized. These cells remain in the blood of autoimmune patients instead of being eliminated and infections may create a favourable environment to activate them, representing a risk to break tolerance and develop autoimmunity.

Taken together, our results give evidence that CR1 efficiently inhibits the BCR-induced functions of human B cells and further supports the indispensable role of complement in regulation of humoral immunity. While triggering via the BCR has been thoroughly investigated [28], modulation of BCR-initiated intracellular signaling by complement receptors has not been studied so far. Our data provide insight for the first time into this inhibitory mechanism by revealing that CR1 ligation decreases phosphorylation of key molecules of the BCR-induced signaling cascade. Since we have proven that the reduced CR1 expression does not influence the physiological function of the receptor, we assume that CR1 may be considered as a potential therapeutical target in certain “B cell-driven” autoimmune diseases, like SLE and RA, to decrease activation of autoreactive B cells.

## Abbreviations

Ab: Antibody
Ag: Antigen
ahIgG+A+M: Anti-human IgG+A+M
BCR: B cell receptor
CR: Complement receptor
ERK: Extracellular signal regulated kinase
IC: Immune complex
JNK: c-Jun N-terminal kinase
MAPK: Mitogen activated protein kinase
RA: Rheumatoid arthritis
SLE: Systemic lupus erythematosus.
